# The role of long intergenic non-coding RNA for kinase activation (*LINK-A*) as an oncogene in non-small cell lung carcinoma

**DOI:** 10.1038/s41598-021-82892-z

**Published:** 2021-02-18

**Authors:** Parichehr Maleki, Seyed Javad Mowla, Mohammad Taheri, Soudeh Ghafouri-Fard, Jamshid Raheb

**Affiliations:** 1 Department of Molecular Medicine, National Institute of Genetic Engineering and Biotechnology, Tehran, Iran; 2 Department of Molecular Genetics, Faculty of Biological Sciences, Tarbiat Modares University, Tehran, Iran; 3 Urology and Nephrology Research Center, Shahid Beheshti University of Medical Sciences, Tehran, Iran; 4 Urogenital Stem Cell Research Center, Shahid Beheshti University of Medical Sciences, Tehran, Iran; 5 Department of Medical Genetics, Shahid Beheshti University of Medical Sciences, Tehran, Iran

**Keywords:** Biotechnology, Cancer, Genetics

## Abstract

The oncogenic role of long intergenic non-coding RNA for kinase activation (*LINK-A*) has been appraised in triple-negative breast cancer. However, the molecular function of *LINK-A* is still unclear in most cancers including lung cancer. The present study aimed to evaluate the impact of down-regulation of *LINK-A* in A549 and Calu-3 cell lines as cellular models of non-small cell lung carcinoma (NSCLC). We used the RNA interference system to knock down *LINK-A*. *LINK-A* expression was significantly reduced by siRNA transfection in A549 and Calu-3 cell lines. *LINK-A* down-regulation significantly reduced cell viability, colony-forming ability and cell migration, as measured by MTT, colony formation and invasion assays. Finally, cell cycle analysis and Annexin-V/7AAD staining indicated that apoptosis was influenced by *LINK-A* silencing. Taken together, *LINK-A* can be proposed as an oncogene in NSCLC.

## Introduction

Cancer is the second leading cause of mortality worldwide^1^. Lung cancer has the highest rank of incidence and global death rate among all cancers at present^2^. Non-small cell lung cancer (NSCLC) comprises approximately 85% of all kinds of lung cancer cases^3^. If being diagnosed in the late stage of the disease, NSCLC patients have a poor prognosis with 17% overall survival rate despite administration of radiotherapy, chemotherapy and other novel therapeutic tools^4,5^. Therefore, identification of molecular mechanisms involved in the pathogenesis of this cancer and recognition of biomarkers for early diagnosis and therapeutic targets are necessary. Long non-coding RNAs (lncRNA) are RNA transcripts longer than 200 nucleotides with limited coding potential, which have received enormous attention in cancer studies in the last years^6,7^. Dysregulation of their expressions influences cell proliferation, metastasis, and invasion of tumor cells^8–10^. They are implicated in a variety of biological processes by regulating gene expression in *cis* or *trans* modes by various mechanisms including epigenetics, transcriptional, and posttranscriptional regulatory mechanisms^11^. Long intergenic non-coding RNA for kinase activation (*LINK-A*) is as an oncogenic cytoplasmic non-coding RNA with approximately 1.5 kbp length. This lncRNA provides a scaffold-like structure in the cytoplasm which interacts with some proteins. In addition, it mechanistically facilitates the recruitment of protein kinase 6 (BRK) to the epidermal growth factor receptor EGFR: GPNMB complex (transmembrane tyrosine receptor and NMB glycoprotein, respectively) and provokes the conformational change of BRK for kinase activation. On the other hand, *LINK-A* interacts with leucine-rich repeat kinase 2 and subsequently causes hypoxia-inducible factor 1 (HIF)-1α phosphorylation and stabilization in normoxic conditions. Ultimately, the consequences of these events are glycolytic reprogramming and tumorigenesis in triple-negative breast cancers cell lines^12^. HIF1α is not exclusively regulated by hypoxia and, in some cases, other stimulators influence its activity even more. Considering its crucial role in cancer progression, HIF1α as a transcription factor is involved in several signaling pathways and overlapping molecular mechanisms, each of which could be an encouraging target to be investigated in cancer studies^13,14^. In addition to its roles in breast cancer, *LINK-A* has oncogenic role in ovarian cancer^15^, glioma ^16^, and lung cancer^17,18^. *LINK-A* acts as a cytoplasmic scaffold in triple-negative breast cancer cell lines (MDA-MB-231 and MDA-MB-468) leads to the normoxic stabilization of HIF1α^12^. Considering the previously known impact of *LINK-A* on the hyperactivation of HIF1α in triple negative-breast cancer, the current study aimed to investigate *LINK-A* function in Calu-3 and A549 cell lines as representative models of NSCLC. More precisely, the study has focused on the role of *LINK-A* in several tumoral features (i.e., cell proliferation, apoptosis, and wound healing) by silencing *LINK-A* with the RNA interference system. It was further aimed to examine Angiopoietin-like protein 4 (ANGPTL4), Basic Helix-Loop-Helix Family Member E40 (BHLHE40), and vascular endothelial growth factor (VEGF) expression alterations as the ultimate targets of HIF1α by relying on the aforementioned correlation between *LINK-A* and the hyperactivity of HIF1α and the consequent probable downstream outcomes.

## Materials and methods

### Cell culture and transfection

The A549 and Calu-3 human lung adenocarcinoma cell lines were obtained from Pasture Institute (Tehran, Iran). The cells were cultured in Dulbecco’s Modified Eagle’s Medium (DMEM, Gibco) supplemented with 10% fetal bovine serum (FBS, Gibco) and 1% penicillin–streptomycin in a 98% humidified atmosphere with 5% CO_2_ incubator (binder) at 37 °C. Two siRNAs hitting the *LINK-A*, Hs-cell death control siRNA as positive control and scrambled siRNA as negative control were purchased from Qiagen Company. *LINK-A* targeting siRNAs were 21 nucleotide length and had 5′-Fluorescein (6 FAM) modification on sense strand for monitoring the efficiency of siRNA delivery into transfected cells by observing under the fluorescent inverted microscope. For siRNA transfection, 4 × 10^5^ cells were seeded in each well of 6-well tissue culture plates one day before transfection. Moreover, transfection was conducted by Lipofectamine 2000 according to the manufacturer’s instructions in reduced FBS (5%) and free antibiotics media. All experiments were conducted in triplicates.

### RNA extraction, cDNA synthesis, and qPCR

After 48 hours from transfection, the total cellular RNA was extracted by the TriPure Isolation Reagent (Roche, Germany) according to the standard procedure defined by the manufacturer’s protocol. Additionally, cDNA was synthesized by RevertAid First Strand cDNA Synthesis Kit (Thermo Fisher Scientific, Inc) using two micrograms of total RNA treated by DNaseI (Thermo Fisher Scientific, Inc). Then, the real-time polymerase chain reaction (PCR) was conducted by the SYBR Green PCR kit (Roche) for the quantitative expression analysis of genes examined in this study with specific primers listed in Table 1. Next, thermal cycling was implemented in the Magnetic Induction Cycler system in the specific scheduled program for each primer pairs.

**Table 1:** Oligonucleotide primers used in real-time PCR.

Link-A F	ACAGCTCATTTATCCATTTTCCTAC
Link-A R	CAGAGATATACACAACAATTTCATACC
ANGPTL4 F	CCACTTGGGACCAGGATCAC
ANGPTL4 R	CGGAAGTACTGGCCGTTGAG
BHLHE40 F	GACCGGATTAACGAGTGCAT
BHLHE40 R	TGCTTTCACATGCTTCAAGG
VEGF F	AACTTTCTGCTGTCTTGGGTG
VEGF R	ATGTCCACCAGGGTCTCGATT
BAX F	CTGACATGTTTTCTGACGGCAA
BAX R	GAAGTCCAATGTCCAGCCCA
BCL2 F	ATTGTGGCCTTCTTTGAGTTCG
BCL2 R	ATCCCAGCCTCCGTTATCCT
GAPDH F	CATCAAGAAGGTGAAGCAG
GAPDH R	GCGTCAAAGGTGGAGGAGTG

Before siRNA transfection, the amplified PCR product of *LINK-A* was purified and cloned into the compatible site of the Ptg19-T PCR cloning vector (Cinnagene Company, IRAN) and then sequenced with M13 forward and reverse primers by BigDye technology on an AB13700 XL sequencer applied biosystem. Finally, the blast program was used to confirm the accuracy of the detected sequence by matching the sequence with the published NCBI sequences for *LINK-A*.

### MTT assay

Cell viability was assayed by 3-(4, 5-dimethylthiazol-2-yl)-2 5-diphenyl-tetrazolium bromide (MTT) colorimetric assay (Sigma-Aldrich, St. Louis, Mo, USA). Briefly, 3 × 10^4^ cells were seeded per well of 96-well plates and, as described previously, transfected with various concentrations of *LINK-A*-siRNAs. After 24 and 48 hours, 10 µl MTT solution (5 mg/ml) was added into the each well and left into the incubator for further four hours in order to complete the reaction. Then, the media was replaced with 100 µl dimethyl sulfoxide (DMSO, Sigma-Aldrich) in order to dissolve formazan crystals, followed by measuring the absorbance of each well using a microplate photometer (LabSystem Multiscan). The test was performed in triplicates.

### Colony formation assay

The colony formation potential of down-regulated *LINK-A* cells was monitored by the routine colony formation assay after 24 hours from transfection. A total of 500 and 200 cells of A549 and Calu-3 cells were seeded in each well of 6-well tissue culture plates with a complete medium, respectively. Cells were maintained under the routine cell culture condition (37 °C, 5% CO_2_, and humidified air) for 7 days by medium replacement every three days. Next, the colonies were washed twice with phosphate-buffered saline (PBS), fixed with cold 95% methanol for 10 minutes, and then stained by 1% trypan blue for 10 minutes. Subsequently, the dye was removed and the plates were washed with PBS before counting and imaging under the microscope. Colonies containing less than 50 cells and smaller than 1 mm were excluded from the counting. The experiment for each group was performed in three replicates.

### Wound healing assay

The two-dimensional cell migration was calculated by the simple wound healing assay. After 24 hours, transfected cells were serum starved (0.5% FBS) for an overnight to minimize cell proliferation. Furthermore, the cells were almost 80–90% confluent when a yellow tip was used for creating scratches across cell monolayers. Then, the cellular debris was removed and washed twice with sterile PBS. Then, fresh media was added into the cells. Moreover, the wounded region was examined and photographed by light microscopy immediately, 24, and 48 hours later. The wound edges and migrated cell numbers in the gap areas for the same fields in the photographs were determined and calculated with ImageJ software I, version 1.4.

### Transwell assay

Transwell chambers with a pore size of 8.0 µm were used to assess cell migration. Briefly, after 24 hours from transfection, 5 × 10^4^ cells were suspended in 250 µl serum free DMEM medium and seeded in the upper chamber of the inserts in the transwell plates (SPL). Then, 750 µl complete medium (10% FBS) was added in the lower chamber as a chemotactic factor. After 48 hours incubation in cell culture condition, cells that were retained on the upper surface of the transwells were wiped off using cotton swab, while the migrated cells to the bottom side were fixed with methanol and stained with Giemsa solution. For invasion assay, the upper 8 µm chambers were coated with Matrigel and dried an overnight in the incubator. The next day, 5 × 10^4^ cells were seeded on the matrigel coated chambers following aforementioned the procedure for the migration assay. The number of penetrating cells was counted in five randomly selected fields in the photographs with 200 magnification taken by an inverted microscope.

### Apoptosis analysis by Annexin V-PE/7AAD

Annexin V-PE/7AAD dual staining was conducted to determine the range of apoptosis in *LINK-A* siRNA transfected cells using the Annexin V-PE (Phycoerythrin) and 7AAD (7-amino-actinomicine D) apoptosis detection kit (eBioscience, Thermo Fisher Scientific) according to the manufacturer’s method. Briefly, 24 and 48 hours after siRNA transfection, the cells were harvested by trypsinization and suspended in PBS 1× and then stained with Annexin V-PE and 7AAD for 15 minutes in the dark at room temperature. This double staining approach allowed the detection of early-stage apoptosis (Annexin-positive cells) and late-stage apoptosis/necrosis (7AAD-positive cells). Additionally, the stained cells were immediately assayed by a BD FACSCalibur flow cytometer (BD Biosciences, San Jose, CA) and the acquired data were analyzed using the FlowJo software (tree stat, INC, Ashland, OR).

### Fluorescent visualization of apoptosis by (AO)/ethidium bromide (ET) staining

Fluorescent staining AO/ET (Sigma-Aldrich) was conducted to visualize apoptosis in the cells with reduced expression of *LINK-A* lncRNA compared to the control groups. The cells were washed with PBS and then stained with 5 µg/ml AO and 5 µg/ml EB in 1X PBS. The stained cells were immediately observed after being washed by PBS and imaged under a fluorescence microscope. The consequence of different dye permeability characteristics into intact cell membranes is considered as the distinction between apoptotic and necrotic and viable cells.

### Cell cycle analysis

For the cell cycle assessment, the transfected cells with siRNAs targeting *LINK-A* (in the pool form and the final concentration of 100 nM) were washed with PBS, harvested and then fixed with 70% cold ethanol. After washing the cells twice with PBS, they were resuspended in the PI/Triton X-100 staining solution including Triton X-100 (0.1% [v/v]), 10 µg/ml propidium iodide, and 100 µg/ml DNase-free RNase A in PBS. The cell cycle analysis was performed on a BD FACSCaliur flow cytometer (BD Biosciences) after 30 minutes of incubating the stained cells in the dark condition at room temperature. Eventually, the percent of the cell population in each phase of the cell cycle 24 and 48 hours after transfection was determined using the FlowJo software (Tree Star, Inc).

## Results

### Significant down-regulation of *LINK-A *in transfected cells with *LINK-A* siRNAs

**Figure 1:**
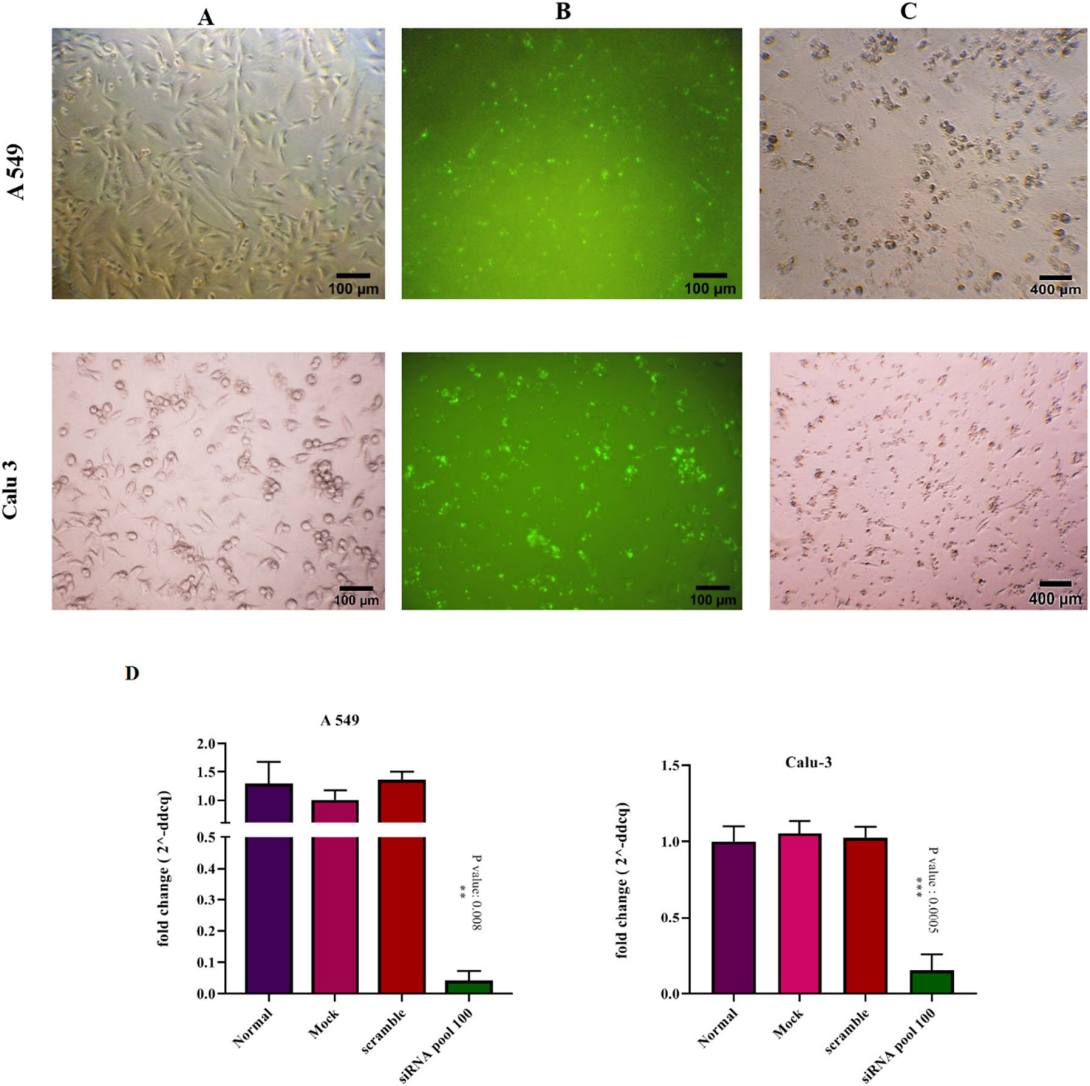
Knockdown of *LINK-A* in A549 and Calu-3 cell lines by the RNA interference system*. Note*. (**A**) The light microscopy images of siRNA transfected A549 and Calu-3 cells. (**B**) The fluorescent microscope image of siRNA transfected cells, which siRNAs were labeled with 5′-Fluorescein 6 FAM tags. Green fluorescent signals indicate efficient siRNA delivery into the cells. (**C**) The light microscope image of cells transfected with Hs cell death control siRNA represents intense cell death after 72 hours, which confirms transfection and down-regulation efficiency. (**D**) The graphs of RNA expression change of *LINK-A* after transfection compared with control groups. The normal group consists untreated cells; Mock controls are cells treated with only Lipofectamin 2000 and the third control group is cells transfected with scrambled siRNA. The control groups were not significantly different from each other (*P* > 0.05). The error bar indicates the mean ± SD.

The real-time PCR approach was utilized to examine endogenous RNA expression in the transfected cells after 48 h of transfection. The efficiency of the transfection was confirmed by observing massive cell death following transfection with HS-cell death control siRNAs as a positive control, along with the experiments. In addition, the expression of the *LINK-A* in the cells transfected with siRNAs targeting *LINK-A* was significantly declined compared with the control groups which included the cells that were transfected with scrambled siRNA or treated only with Lipofectamine 2000 (Mock group). There was no significant difference between the mock group and scrambled-siRNA transfected cells. *LINK-A* siRNAs were fluorescent-labeled (5′-Fluorescein 6 FAM) to determine efficient siRNA delivery into the cells and Student’s t-test analysis was conducted to evaluate statistically significant differences (Fig. 1).

### The significant role of *LINK-A* suppression in the inhibition of proliferation and the colony formation of A549 and Calu-3 cancerous cell line

**Figure 2:**
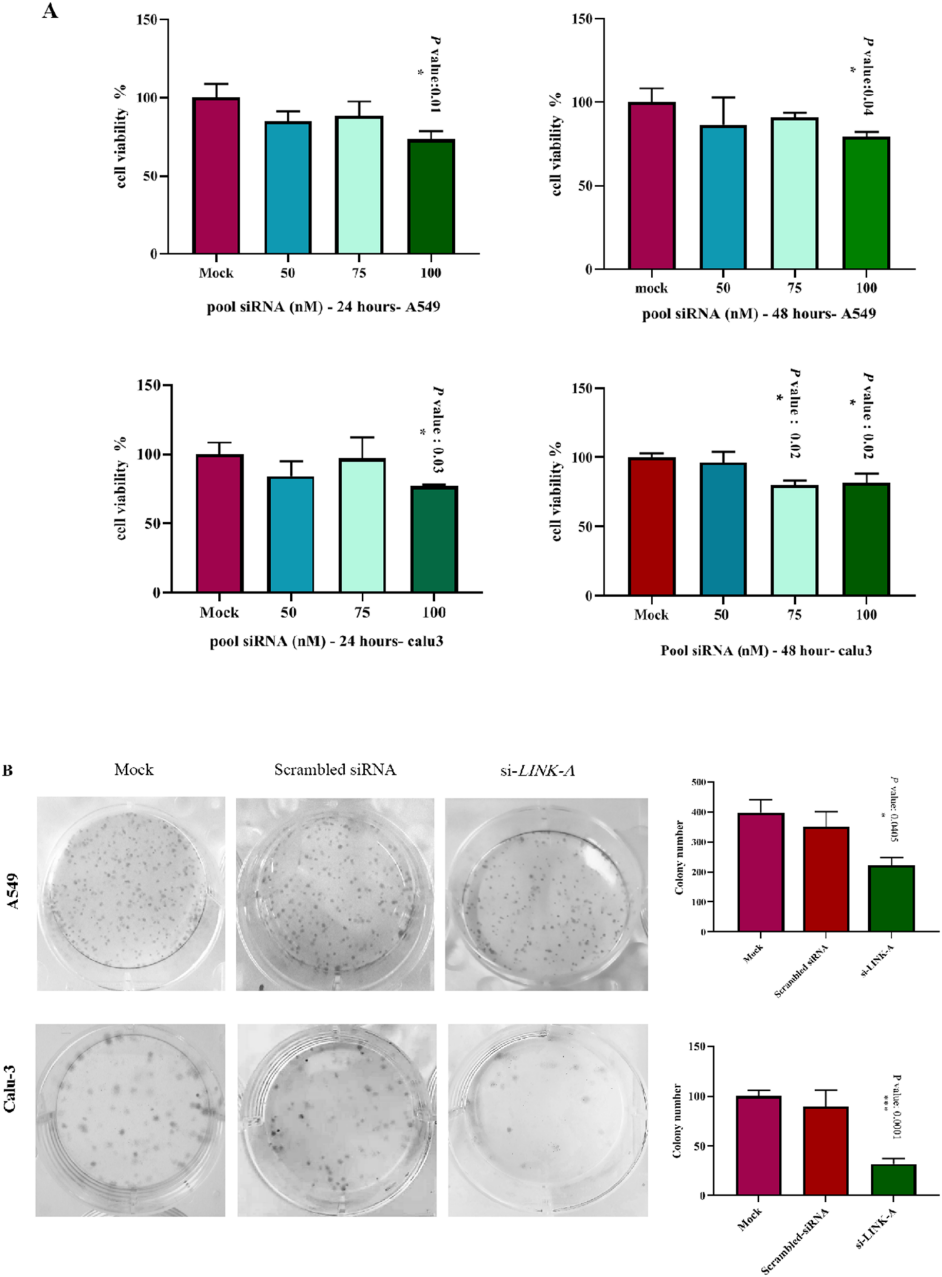
*LINK-A* downregulation impacts on cell viability and proliferation. (**A**) The Graphs of MTT assays in 24 and 48 hours after transfection in A549 and Calu-3 cell lines, the error bars show mean ± SD (*P value < 0.05, ***P value < 0.001, two-tailed t test). (**B**) Colony formation assay images represent reduced colony-forming ability in *LINK-A* silenced cells compared to control groups. The error bar indicates the mean ± SD. (*P value < 0.05, two-tailed t test).

The MTT and colony-forming assay were conducted to investigate cell viability and proliferation rate influenced by *LINK-A* down-regulation. In this study, different concentrations of siRNAs were used in the pool form for the MTT assay and investigated in 24 and 48 hours after transfection. In the A549 cell line, only the concentration of 100 nM siRNAs significantly impacted cells viability in 24 and 48 hours after transfection (*P < 0.05), however in the Calu-3 cell line, the concentration of 100 nM siRNAs in 24 hours and 75 nM as well as 100 nM in 48 hours later from transfection reduced cell viability significantly (*P < *0.05). LINK-A* contribution in colony-forming ability was confirmed by using pool 100 nM concentration of siRNAs as well (Fig. 2). The cells with suppressed *LINK-A* expression exhibited a dramatic decrease in the size and number of colonies seven days after transfection (*P < 0.05, ***P < 0.001 in A549 and Calu-3 cell lines, respectively by two-tailed t-test).

### Inhibition of the metastatic potential of A549 and Calu-3 cells by down-regulation of *LINK-A* lncRNA

**Figure 3:**
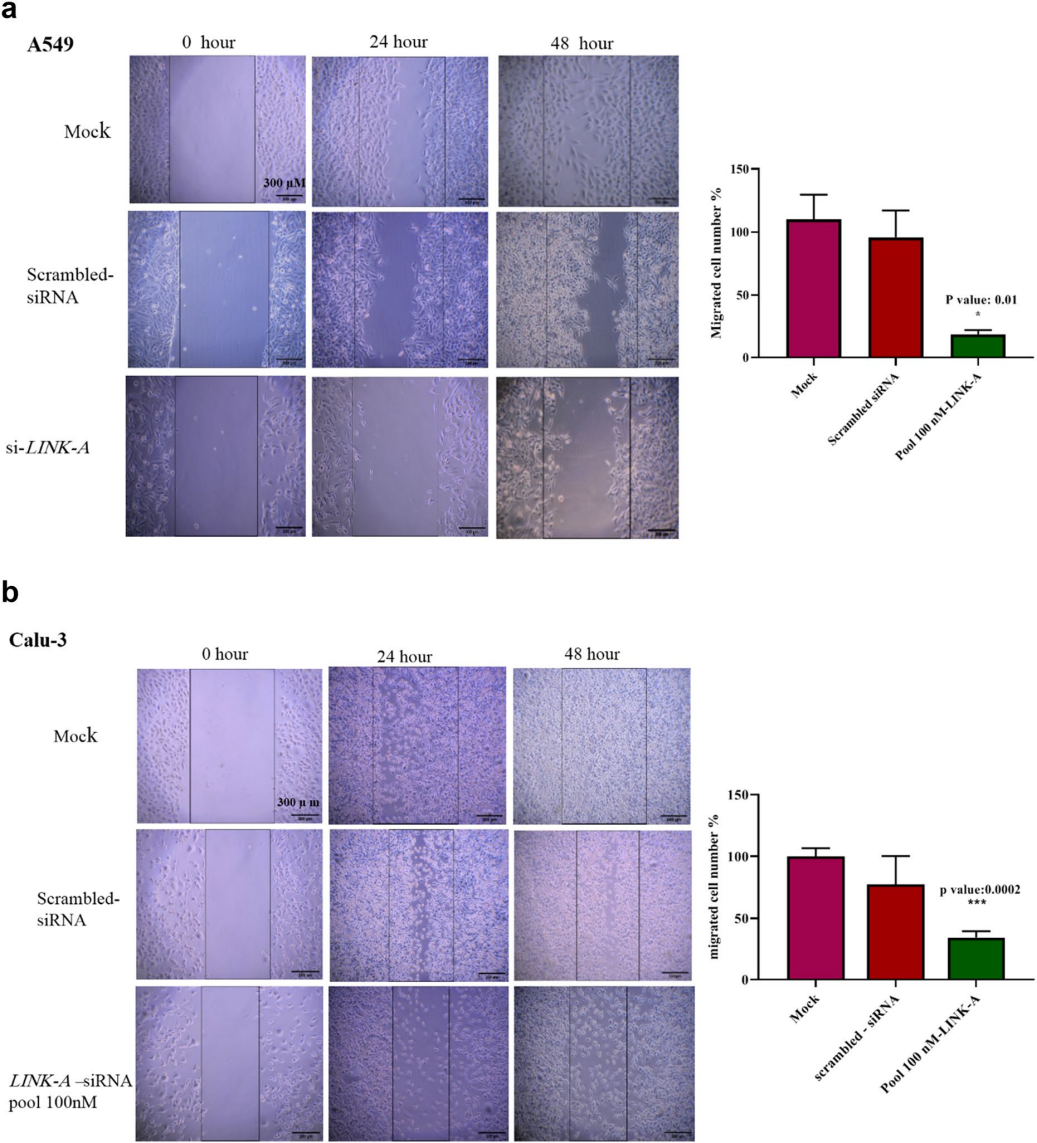
*LINK-A* knockdown effect on mobility of A549 (**A**) and Calu-3 cell lines (**B**) as assessed by the wound healing assay. *Note*. Images of scratched control cells (mock, scrambled siRNA transfected cells) and *LINK-A* siRNA (pool 100 nM) transfected cells in time points of 0, 24, and 48 hours after creation of scratch with objective magnification 10×. The wound edges were delineated by black lines and the numbers of migrated cell numbers in the wound areas were counted for each field by ImageJ software. The graphs are presented as the mean ± SD (*P < 0.05, ***P < 0.001).

Cell migration plays a critical role in cancer progression and metastasis, thus in the present study, the wound healing assay was performed to investigate the possible impact of *LINK-A* on the motility and migration capability of A549 (Fig. 3A) and Calu-3 cells at a two-dimensional surface (Fig. 3B).

**Figure 4:**
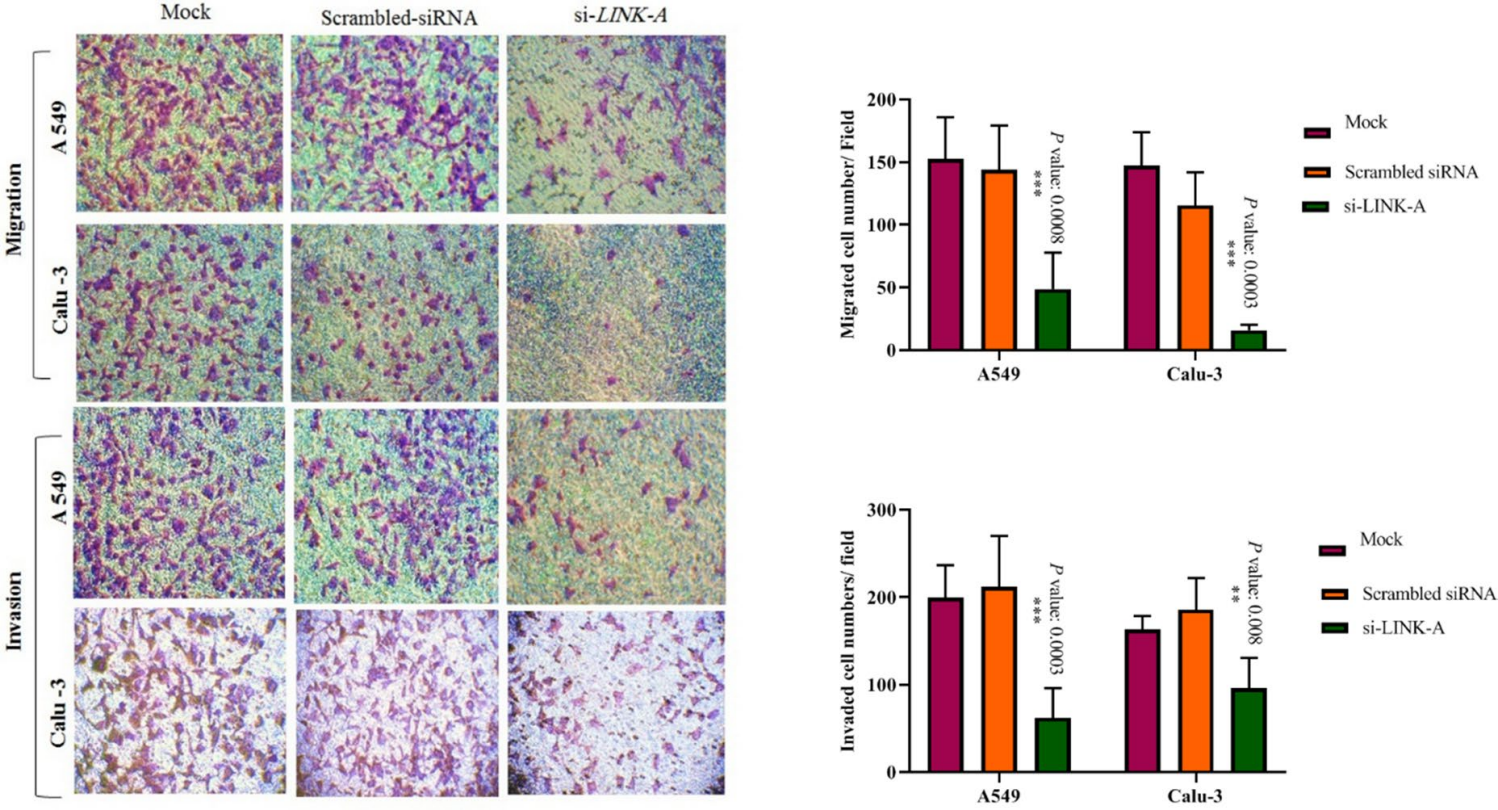
Effect of down-regulation of *LINK-A* on migration and invasion of A549 and Calu-3 cell lines using transwells. *Note.* Transwell and matrigel coated transwells were used to determine *LINK-A* suppression effect on migration and invasion ability of cells compared with controls for each cell line. si-*LINK-A* are cells transfected with pool 100 nM *LINK-A* siRNA, which were seeded on transwell inserts 24 hours later from transfection. Traversed cells at the bottom side of the chambers were photographed with 200× magnification after 48 hours and were counted in 5 different random fields. Data are presented as mean ± SD (***P value < 0.001).

A remarkable decline of migrated cell numbers in the wound gaps was observed for the *LINK-A* down-regulated Calu-3 (***P = 0.0002), and A549 cells (*P = 0.01). In addition, we conducted migration and invasion assay using transwells and matrigel coated transwells, respectively, which indicated significantly reduced migration and invasion capability of investigated cells influenced by *LINK-A* down-regulation (Fig. 4). Therefore, these results represent the probable function of *LINK-A* in cell mobility or invasion, emphasizing the precise evaluation of this topic in future studies.

### *LINK-A* silencing effects on cell cycle regulation

**Figure 5:**
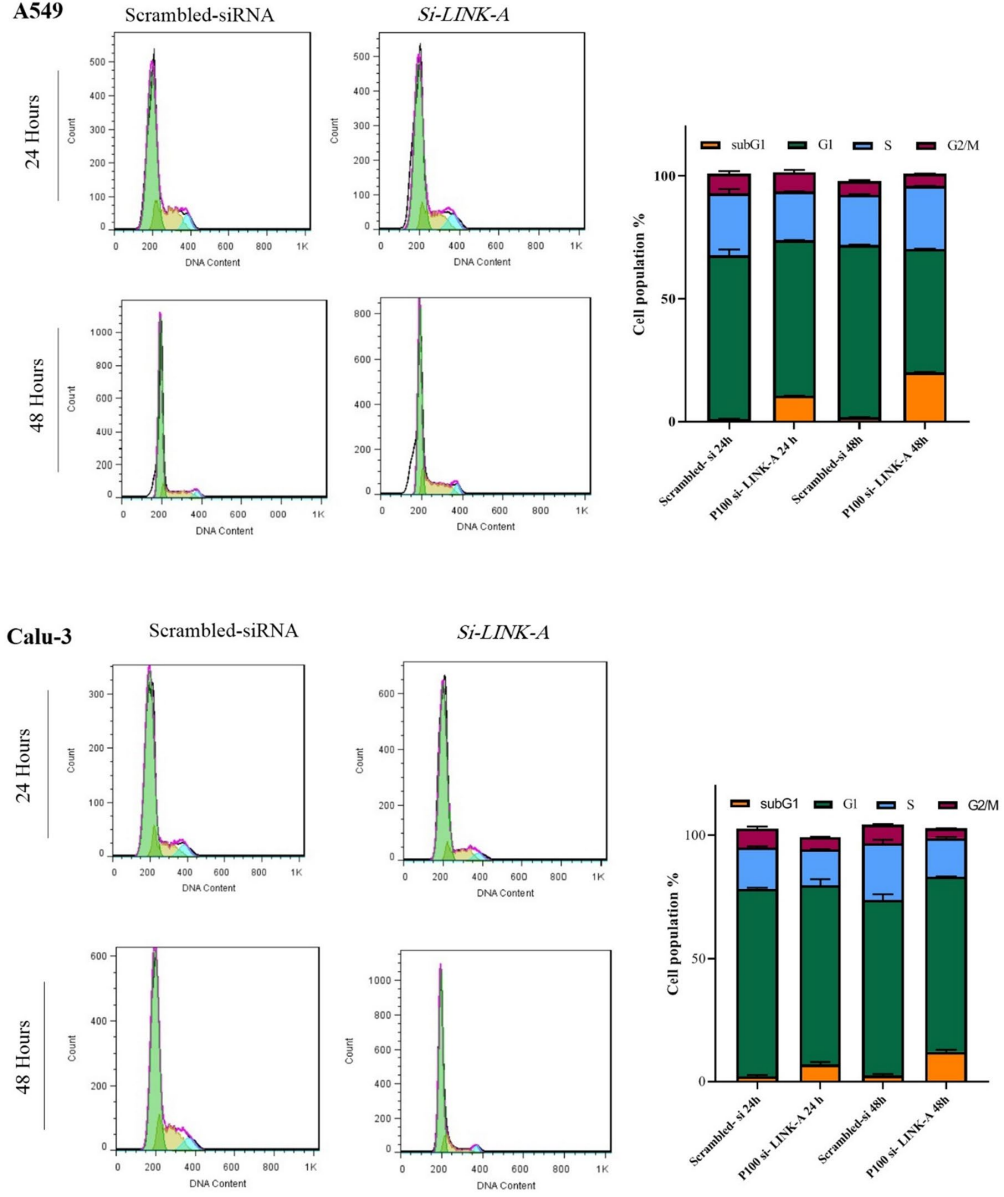
Flow cytometric analysis of cell cycle by PI staining in *LINK-A* silenced cells. *Note.* Cell cycle analysis after 24 and 48 hours from transfection with scrambled siRNA (as control groups) and *LINK-A* siRNAs in A549 and Calu-3 cell lines. The graphs are presented for each cell line separately, which show the distribution of the cell population in various phases of the cell cycle. Data are presented as the mean ± SD.

Flow cytometric analysis by propidium iodide was performed after *LINK-A* siRNA transfection (pool 100 nM) to determine whether the observed *LINK-A* effect on cell viability was due to the inhibition of cell proliferation or the elevation of cell death. The obtained data represents the significantly increased percentage of the sub-G1 phase in *LINK-A* silenced cells compared with the control groups for both A549 and Calu-3 cells. Two-way ANOVA analysis was used by GraphPad Prism 8.0.2 software to determine the cell population differences between sub-G1 phases of *LINK-A* suppressed and control cells for each cell line and time point (****P < 0.0001 in 24 and 48 hours after transfection for A549, *P < 0.05 in 24 hours, ***P < 0.001 in 48 hours after transfection for Calu-3 cell line). G2/M demonstrated no significant difference between the knocked-down *LINK-A* and control A549 cells, however it was significantly reduced in the *LINK-A* silenced Calu-3 cells 48 hours after transfection (Fig. 5).

### Induction of apoptosis by *LINK-A* silencing

**Figure 6:**
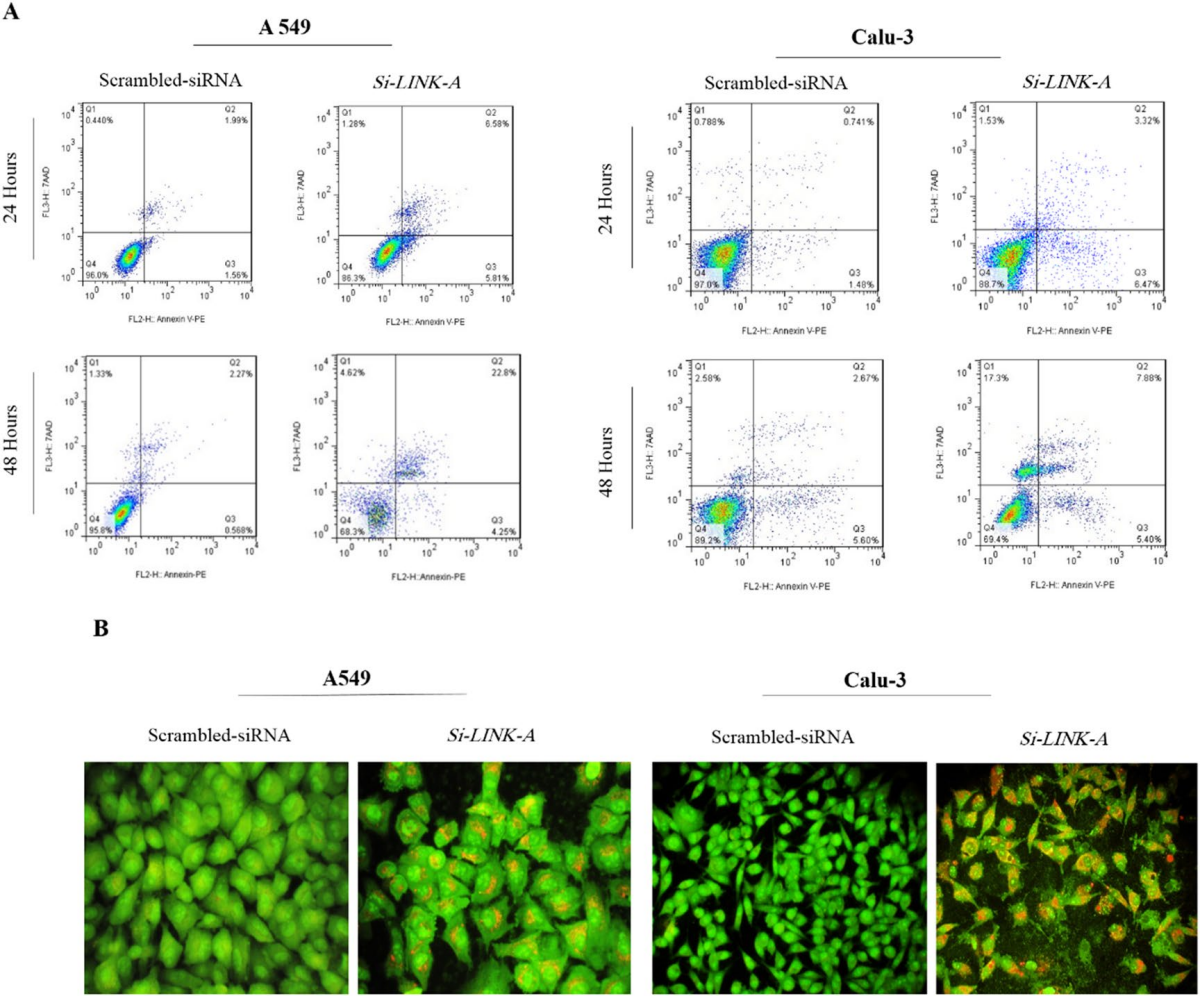
Induction of cell death by suppression of *LINK-A*. *Note*, A. apoptosis assay by annexin V-PE/ 7AAD and flow cytometry in 24 and 48 hours after transfection in A549 and Calu-3 cell lines. In the graphs, quarters of Q_1_, Q_2_, Q_3_, and Q_4_ represent dead cells, late apoptosis/necrosis, early apoptosis, and live cells, respectively. B. Acridine orange and ethidium bromide staining in a549 and calu-3 cell lines performed 48 hours later from transfection.

Based on cell cycle analysis, the silencing of *LINK-A* affected cell viability, proliferation and caused cell death. Furthermore, Annexin-V-PE/7AAD staining was conducted to determine *LINK-A* role in the apoptosis and necrosis. The obtained data from A549 revealed an elevated rate of late apoptotic cells in *LINK-A* siRNA transfected cells (pool 100 nM) related to the controls. In the A549 cell line, the late apoptotic cells were 1.99% and 6.58% in the control and *LINK-A* silenced groups 24 hours after transfection, respectively. Moreover, the extent of the late apoptotic cell was increased from 2.27 to 22.8%, 48 hours after transfection. The rate of the late apoptotic cells in the Calu-3 were 0.74% and 3.32% in the control and *LNK-A* down-regulated cells, 24 hours later from transfection, respectively. Furthermore, 48 hours after transfection, the percentage of late apoptotic cells was almost doubled compared to the control group. We also observed 17% cell death in the *LINK-A* silenced Calu-3 cells (Annexin-v negative, while 7AAD positive). Additionally, acridine orange (AO) and ethidium bromide staining showed occurrence of apoptosis in *LINK-A* down-regulated cells and obvious cellular morphology differences between control and *LINK-A* siRNA treated cells (Fig. 6).

**Figure 7:**
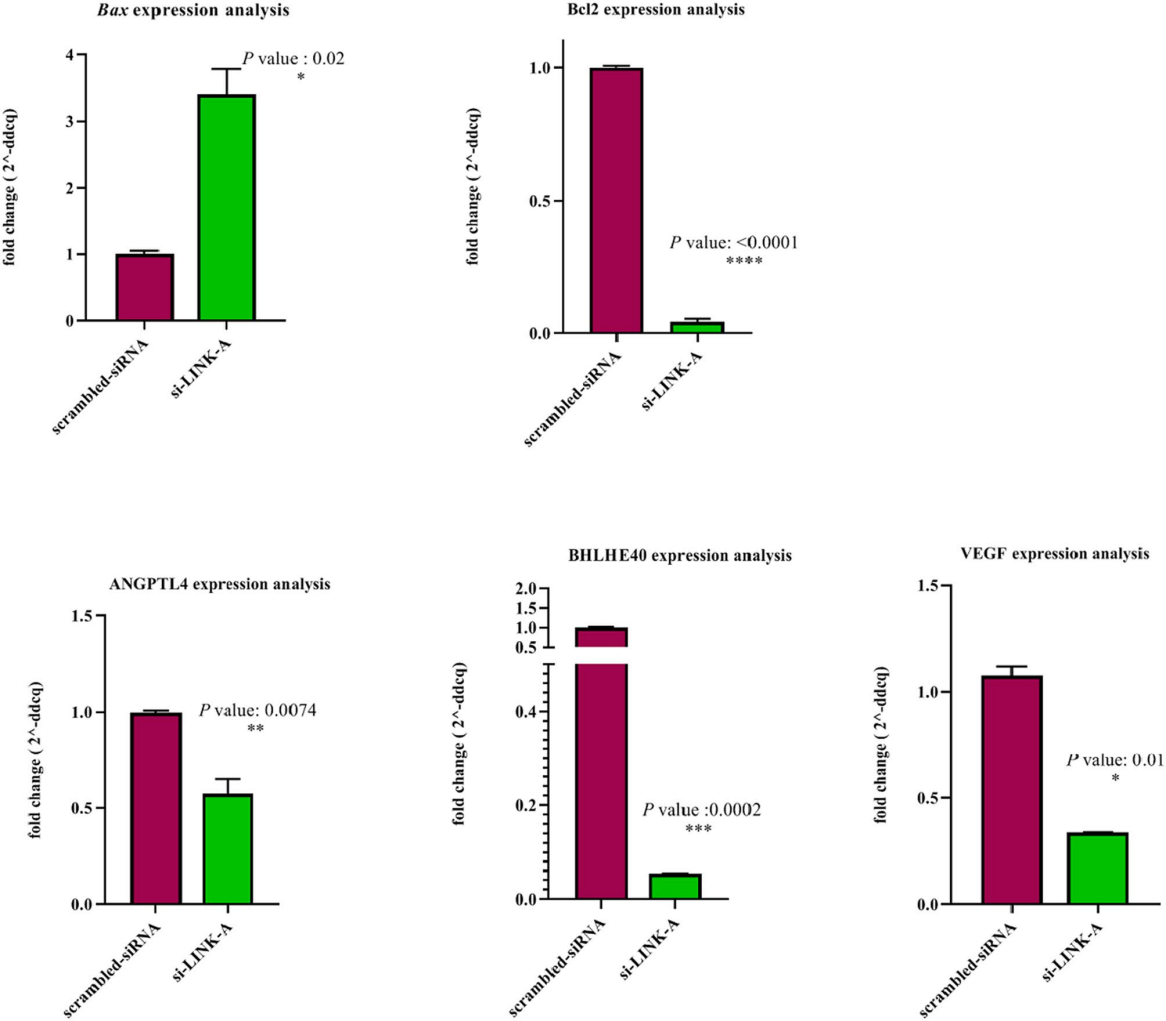
Graph bars of Real time PCR. RNA expression changes of *VEGF*, *ANGPTL4*, *BHLHE40*, *BAX*, *BCL2* in *LINK-A* down-regulated A549 cells. Data are presented as mean ± Sd. Two-tailed student’s t-test analysis was performed for each gene separately (*P < 0.05, **P < 0.01, ***P < 0.001).

The green cells with the normal morphology of the nucleus represent the living cells which are stained only with AO. In addition, the green and orange cells with condensed chromatin and blebbing cell membrane characterize apoptotic cells and are stained with both AO/ET because of a moderate alteration in cell membrane permeability. The present study also examined the RNA expression analysis of *Bax* and *Bcl2* genes involved in cell death. The findings showed that the transfection of A549 cells with siRNAs hitting *LINK-A* causes significant up-regulation of *Bax* (*P = 0.0234) and noticeable down-regulation of *Bcl2* expression (****P < 0.0001). The graphs are shown in Fig. 7.

### Altered mRNA expression of BHLHE40, ANGPTL4, and VEGF

Considering earlier discoveries, which highlight the influence of *LINK-A* lncRNA on some genes and pathways, the study examined BHLHE40, ANGPTL4, and VEGF expressions after the silencing of *LINK-A*. The real-time PCR was conducted in *LINK-A* siRNA transfected cells (100 nM Pool siRNA) and the acquired data indicated noticeable down-regulation for BHLHE40 (***P = 0.0002) and ANGPTL4 (**P = 0.0074), along with the reduced expression of VEGF (*P = 0.01) (Fig. 7).

## Discussion

The findings of the present study revealed that *LINK-A* down-regulation could induce apoptosis and reduce cell survival and proliferation in the A549 and Calu-3 cell lines. Moreover, the induction of apoptosis was confirmed by the cell cycle and Annexin-V/PI assay with flow cytometry, as well as acridine orange and ethidium bromide staining. The quantification of the mRNA expression levels of apoptosis-related genes in *LINK-A* silenced cells indicated significant up- and down-regulation of *Bax* and *Bcl2*, respectively. High *Bcl2* expression levels have been reported in a variety of cancers^19–21^. *Bcl2* plays a vital role in protecting cancerous cells and fosters the progression of cancer by regulating apoptosis^22^, while *Bax* acts as a tumor suppressor in human malignancies through mediating the apoptosis in response to genotoxic stresses^23^. The impact of *LINK-A* silencing on the expression of these crucial genes and the consequent induction of apoptosis propose the oncogenic role of *LINK-A* in the A549 and Calu-3 cell lines and designate *LINK-A* as a probable therapeutic potential in lung cancer. This study did not scrutinize the molecular mechanism of *LINK-A* contribution to the regulation of *Bcl2* and *Bax* expressions. However, it is supposed that *LINK-A* acts as a mediator between the signaling pathways, therefore, the alteration of expression patterns probably is the consequence of multiple events concerning the indirect upstream system of nuclear expression regulatory machine. In addition, Lin et al. have informed the antiapoptotic function of *LINK-A* lncRNA in MDA-MB-231 of the breast cancer cell line^24^. Considering these observations, this study was the first one to propose that *LINK-A* lncRNA has a role in the regulation of apoptosis in A549 and Calu-3 cell lines as representatives for NSCLC. Based on the findings of the present study and previous knowledge about *LINK-A* and HIF1α hyper-activation, a similar function was supposed for *LINK-A* in lung cancer and the RNA expression change of some imperative genes which could be influenced by the silencing of *LINK-A* was examined. ANGPTL4, BHLHE40, and VEGF are considered as critical genes in cancer studies, which play pivotal roles in biological processes and are somehow regulated by HIF1α. Further, the RNA expression alteration of the mentioned genes was evaluated by the real-time PCR and the findings represented that all genes were significantly down-regulated in *LINK-A* siRNA transfected cells. ANGPTL4 belongs to the VEGF family and is structurally related to the proteins modulating angiogenesis known as angiopoietins (ANG). This protein has manifold roles such as lipid metabolism, angiogenesis, cell differentiation, tumorigenesis, and metastasis^25^. Zhu et al. have demonstrated that ANGPTL4 is associated with NSCLC prognosis and its down-regulation could impede tumor cell proliferation and migration ability by the extracellular signal-regulated kinase signaling pathway. They have also found the significant up-regulation of ANGPTL4 in NSCLC tissues related to non-tumor tissues^26^. The potential pro-metastatic function for ANGPTL4 was confirmed in breast cancer^27^, hepatocellular carcinoma cells^28^, and colorectal cancer^29^. In contrast, some studies have found no precise function for ANGPTL4 in cancer progression or even some of them discovered that the elevated expression of ANGPTL4 prevents tumor growth, invasion, and angiogenesis in melanoma and colorectal cancers^30^. HIF1-α is considered as one of the regulatory transcription factors of ANGPTL4 and hypoxia is one potential stimulator for the tumorigenic function of ANGPTL4^31–33^. Accordingly, the present study evaluated the expression change of ANGPTL4 after *LINK-A* down-regulation as a probable ultimate target and the findings revealed a significant decrease. The alteration of the RNA expression of BHLHE40, as another HIF1α target gene, was examined in this study. BHLHE40 role in cancer is unclear although some studies reported the activation of its expression directly by HIF1α in a variety of cancer cells^34,35^. Furthermore, the elevated expression of BHLHE40 was correlated with hypoxia activation, increased metastatic potential, and poor prognosis of various cancer types including hepatocellular carcinoma, pancreatic cancer, and invasive breast cancer^36–38^. Similarly, Sethuraman et al. have discovered that BHLHE40 activates HB-EGF transcription and therefore facilitates cell survival and metastasis progression in breast tumor cells. They have also confirmed that HB-EGF plays a critical role in promoting cell survival and migration^39^. On the other hand, HB-EGF is the key initiating factor of *LINK-A* involved signaling pathway, which leads to the hyper-activation of HIF1α. Moreover, it was found that the down-regulation of BHLHE40 is one of the consequences of *LINK-A* silencing. Based on these findings, it could be hypothesized that *LINK-A* down-regulation probably impedes the trigger of this pathway indirectly and this is a bonus impact of *LINK-A* silencing on preventing the hyper-activation of HIF1α and adverse downstream outcomes. Likewise, Lin et al. have reported significantly reduced RNA expression for *ANGPTL4* and *BHLHE40* in MDA-MB-231 cell lines treated with *LINK-A* targeting siRNAs and HB-EGF factor^12^. VEGF is a predominant angiogenic factor, which is overexpressed in an array of malignancies. The circulating amounts of VEGF are upraised in various cancer patients, including those suffering from lung cancer^40,41^ and the overexpression of VEGF has an adverse influence on the survival of patients with NSCLC^42,43^. HIF1α is one of the multiple inducers of VEGF production, which binds to its regulatory promoter region and elevates VEGF transcription^44^. Based on the findings of this study, significantly reduced expression of VEGF mRNA was observed in *LINK-A* down-regulated cells. This result is important considering the importance of VEGF in the angiogenesis and progression of cancer. Accordingly, future studies are recommended to investigate *LINK-A* and angiogenesis correlation deeply.

Lin et al. have found another remarkable oncogenic role of *LINK-A* in the hyper-activation of AKT and conferring resistance to targeted therapy against the AKT pleckstrin homology domain. The direct interaction of *LINK-A* with PIP3 (phosphatidylinositol-3,4,5-trisphosphate) and Akt facilitates the AKT-PIP3 interface and subsequent AKT activation, which leads to tumorigenesis and extensive clinical implications^24^. According to Wu et al., *LINK-A* is up-regulated in human glioma cells and LDH-A is regulated by *LINK-A*, which mediates the proliferation and invasion of glioma cells^16^. In addition, Zhang et al. have recently reported that *LINK-A* is higher in ovarian carcinoma patients compared with healthy controls, which leads to the metastasis of ovarian carcinoma by up-regulation of HIF1α^15^. In another study conducted by our group, we observed elevated expression of *LINK-A* in Iranian epithelial ovarian cancer patients, which also was correlated with higher stages and grades of cancer^45^. Zhao et al. have reported increased expression levels of *LINK-A* in NSCLC tissues compared with normal tissues. Besides, they have indicated its positive correlation with clinicopathologiacal features and the survival rate of NSCLC patients. Moreover, they have demonstrated that *LINK-A* promotes proliferation of NSCLC cells^18^, which is consistent with our results. In another study conducted by Liu et al., overexpression of LINK-A in metastatic NSCLC has been reported. They have observed that NSCLC patients with higher expression of *LINK-A* had worse survival rates and higher mortality. They have suggested that LINK-A has a role in the promotion of migration and invasion ability of NSCLC cells (H1993, H1581) by activating AKT signaling^17^*.* We also indicated that reduction of *LINK-A* expression in A549 and Calu-3 cell lines significantly inhibited their migration and invasion ability. All these results emphasize the importance of *LINK-A* in NSCLC. *LINK-A,* as a novel lncRNA with oncogenic function in multiple cancers through interaction with various molecular pathways, should be precisely appraised for a therapeutic perspective. This study aimed to provide an overview of the *LINK-A* role in A549 and Calu-3 as NSCLC cell lines in order to find a clue for future works in this research area. In general, the findings indicated the tumorigenic role of *LINK-A* in these cell lines.

## Conclusion

Our observations obtained by the down-regulation of *LINK-A* in two cell lines as a representative model for lung cancer suggest an oncogenic function for *LINK-A*. Reduced expression of *LINK-A* significantly decreased cell proliferation, viability, migration, and invasion abilities. Also, we detected the occurrence of apoptosis following by *LINK-A* down-regulation.
